# Associations between diet, physical activity and body fat distribution: a cross sectional study in an Indian population

**DOI:** 10.1186/s12889-015-1550-7

**Published:** 2015-03-24

**Authors:** Liza Bowen, Amy E Taylor, Ruth Sullivan, Shah Ebrahim, Sanjay Kinra, KV Radha Krishna, Bharati Kulkarni, Yoav Ben-Shlomo, Ulf Ekelund, Jonathan CK Wells, Hannah Kuper

**Affiliations:** St George’s University, London, UK; School of Experimental Psychology, University of Bristol, Bristol, UK; Department of Non Communicable Disease Epidemiology, London School of Hygiene & Tropical Medicine, London, UK; Clinical Division, National Institute of Nutrition, Hyderabad, India; School of Social and Community Medicine, University of Bristol, Bristol, UK; MRC Epidemiology Unit, University of Cambridge, Cambridge, UK; Department of Sport Medicine, Norwegian School of Sport Sciences, Oslo, Norway; Childhood Nutrition Research Centre, UCL Institute of Child Health, London, UK; Department of Clinical Research, London School of Hygiene & Tropical Medicine, London, UK

**Keywords:** Obesity, Body fat, Diet, Physical activity, India

## Abstract

**Background:**

Obesity is a growing health problem in India and worldwide, due to changes in lifestyle. This study aimed to explore the independent associations between dietary and physical activity exposure variables and total body fat and distribution in an Indian setting.

**Methods:**

Individuals who had participated in the Indian Migration Study (IMS) or the Andhra Pradesh Children And Parents' Study (APCAPS), were invited to participate in the Hyderabad DXA Study. Total and abdominal body fat of study participants was measured using DXA scans. Diet and physical activity (PA) levels were measured using questionnaires.

**Results:**

Data on 2208 participants was available for analysis; mean age was 49 yrs in IMS, 21 yrs in APCAPS. Total energy intake was positively associated with total body fat in the APCAPS sample: a 100 kcal higher energy intake was associated with 45 g higher body fat (95% CI 22, 68). In the IMS sample no association was found with total energy intake, but there was a positive association with percent protein intake (1% higher proportion of energy from protein associated with 509 g (95% CI 138,880) higher total body fat). Broadly the same pattern of associations was found with proportion of fat in the abdominal region as the outcome. PA was inversely associated with total body fat in both populations (in APCAPS, one MET-hour higher activity was associated with 46 g (95% CI 12, 81) less body fat; in the IMS it was associated with 145 g less body fat (95% CI 73, 218)). An inverse association was observed between PA and percentage abdominal fat in the IMS but no association was seen in the APCAPS population.

**Conclusions:**

In this Indian population, there was an inverse association between PA and body fat. Associations between body fat and dietary variables differed between the younger APCAPS population and older IMS population. Further longitudinal research is needed to elucidate causality and directions of these associations across the life course.

**Electronic supplementary material:**

The online version of this article (doi:10.1186/s12889-015-1550-7) contains supplementary material, which is available to authorized users.

## Background

Obesity is an escalating health problem worldwide [1]. The National Family and Health Survey of India reported rises in overweight and obesity (BMI ≥ 25) in 15–49 year old women from 7.3% in 1998–9 to 11.1% in 2005–6 [2,3]. As a risk factor for many chronic diseases, notably diabetes, cardiovascular disease, hypertension, and cancers, obesity is a major contributor to the chronic disease burden [4]. Abdominal fat in particular has been shown to be associated with diabetes and cardiovascular disease development [5-10], and Indian populations have a tendency to accumulate fat in the abdominal region [11-14].

Both dietary factors and physical activity patterns are associated with weight gain, though the underlying mechanisms are still being elucidated. Weight can be stored as lean mass or fat mass, and an understanding of which factors favour fat mass deposition could help in obesity prevention. The composition of the diet may be influential; if so, nutrient content as well as total dietary energy intake is important in prevention efforts. Observational and intervention studies assessing associations of obesity with particular nutrients have shown varied results. Some studies report an inverse association between body fat and high carbohydrate diets [15,16], others a positive association between body fat and high fat diet [15,17,18], while both positive and inverse associations have been found between body fat and high protein diets [19-21]. The lack of consistency seen across studies in different settings may be due to a combination of residual confounding factors and the measurement error inherent in self-reported dietary intake. There are also likely to be many unpublished null analyses on associations between dietary factors and body fat, as is true for other areas of research.

Data from the Food and Agriculture Organisation (FAO) show an increase in total energy intake, protein intake and fat intake in India over the last half century, although consumption still falls far below levels in the UK [22]. For physical activity there are no nationwide surveys of behaviours, but studies restricted to certain states repeatedly show that levels of activity are higher in rural than in urban areas, and that occupational activity is a more significant contributor than leisure activity [23,24].

It is generally accepted that physical activity is protective against the development of obesity. However, the relationship between physical activity and weight gain over time is often weak, and may be influenced by confounding, measurement error, and potential reverse causality or bidirectional effects [25]. Physical activity also varies in intensity, and different intensities may have different associations with body fat and distribution [26]. One possible reason for this is that different intensities of activity have different effects on metabolic processes that in turn influence an individual’s propensity to put on weight [27]. In addition, moderate/vigorous physical activity may not lead to weight loss overall, but may be associated with increase in lean mass instead of fat mass, and also changes in body fat distribution [28,29]. Sedentary behaviour may be independently associated with body fat. TV viewing is one activity that has been used as a marker of sedentary behaviour, and has been found to be associated with body fat [30-35]. The ability to fully elucidate the potential intermediary role of dietary and other physical activity behaviours is limited by the measurement error inherent in diet and physical activity estimates, making residual confounding an important possibility.

The contribution of diet composition and physical activity to body fat is important to consider in Indian populations, where obesity and particularly abdominal obesity are growing problems. The objectives of this study were to explore the associations of both diet composition and physical activity with total body fat and the proportion of fat distributed abdominally.

## Methods

### Ethics statement

Ethical approval was obtained from the London School of Hygiene & Tropical Medicine, the Indian Council of Medical Research, the National Institute of Nutrition and the Krishna Institute of Medical Sciences. Informed written consent was obtained from all participants.

### Study design and participant selection

**Figure 1:**
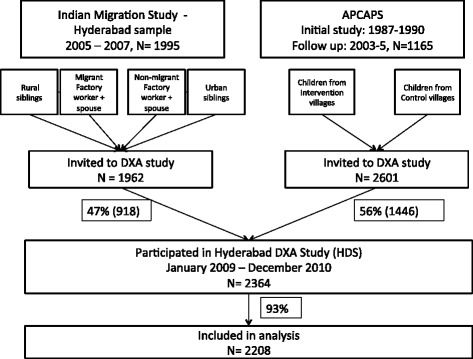
**Flow chart for participation in Hyderabad DXA Study 2009–2010 and inclusion in analysis.**

The Hyderabad DXA Study (HDS) sample population was drawn from two established on-going cohort studies (Figure 1). The first was the Hyderabad arm of the Indian Migration Study (IMS). The IMS is a sibling pair comparison study, recruiting factory workers and their spouses. All rural–urban migrants were invited, along with their siblings still residing in rural areas. A random 25% sample of non-migrants was also invited, along with their urban siblings. Participants were aged 18–79 during IMS data collection (2005–8) [36]. Participants from the Hyderabad arm of the IMS were contacted and invited to take part in the HDS. The second study was the Andhra Pradesh Children And Parents' Study (APCAPS) [37]. APCAPS is a population followed after a nutrition trial conducted in 1987–1990, in which over 2000 women in 29 villages surrounding Hyderabad were randomised (by village) to receive supplementation during pregnancy and the first five years of their child’s life, or to no supplementation [38]. These children, their parents and siblings now form the APCAPS population. Between 2003 and 2005 1165 of the APCAPS children attended a research clinic. All of the eligible children were again approached in 2009–10 and invited to participate in the HDS.

### Measurements

Data collection took place from January 2009 – December 2010. Participants completed an interviewer-administered clinical questionnaire collecting information on age, sex, date of birth, family structure, educational attainment, household circumstances, health and lifestyle. Socioeconomic position was assessed using a subset of 14 of 29 questions from the Standard of Living Index (SLI) [39].

Diet was assessed by an interviewer-administered semi-quantitative food frequency questionnaire (FFQ), adapted from the IMS questionnaire. The development and validation of the IMS FFQ is described in detail elsewhere [40]. Because the IMS FFQ was designed for 4 different regions of India, it was modified to a shorter version appropriate for the Hyderabad region. Contributory and stepwise regression analyses were used to identify foods important in predicting individual nutrient intake. The food items that explained 90% of the between person variability and 90% of contributions to individual nutrient intake were considered for inclusion in the questionnaire, resulting in a list of 98 items. Nutrient databases developed in the IMS were used to calculate nutrient and food group intake. The nutrient information for these came from the Indian food composition tables. For food items where data were not available, the United States Department of Agriculture nutrient database (USDA, Release No.14) or McCance and Widdowsons Composition of Foods was used [41,42].

An interviewer-administered questionnaire was used to assess physical activity undertaken in the week preceding the clinic, based on the validated questionnaire from the IMS [43]. It assessed multiple domains, including household chores, work, travel, sleep, discretionary leisure time, and time spent sedentary. For each activity the average amount of time spent on the activity and the frequency of the activity were documented. Physical activities reported within the HDS were assigned a metabolic equivalent value (MET) using the Compendium of Physical Activity and WHO/FAO/UN guidelines, supplemented with country specific values. One MET is equivalent to approximately 3.5 mL of O_2_/kg/min, or 1 kcal/kg/hour, corresponding to the resting metabolic rate of sitting quietly [44-46]. When all the activities reported did not cumulatively account for 24 hours, a standard MET of 1.4 was applied to the residual time. For manual occupational activity an integrated energy index (IEI) of the activity was applied instead of the absolute MET value. IEI takes into account “rest” or “pause” periods, which individuals are likely to take when engaged in these manual activities. Time (total time [min/day]) spent in categories of activity intensity were generated using previously published cut-points; sedentary ,1.5 MET; light 1.5 to 3 MET; moderate 3 to 6 MET; vigorous >6 METs [47]. Due to the small numbers of participants involved in vigorous physical activity we were unable to consider vigorous activity as a variable. The moderate and vigorous physical activity categories were therefore combined into a single variable: moderate and vigorous physical avtivity (MVPA).

Participants underwent whole body dual energy X-ray absorptiometry (DXA) scans on a Hologic DXA machine (Discovery A model, 91% of scans) or a Hologic QDR 4500 Elite machine (9% of scans). The whole body scan was performed with the participant supine on the scanning bed with their arms resting by their sides to provide measures of total body fat (g). Abdominal fat measures were calculated for the L1 - L4 region: from the midpoint of the intervertebral space between the T12 and L1 vertebrae to the midpoint of the L4 and L5 vertebrae. This region was drawn onto the whole body scan using the Hologic software by a single technician. This procedure was carried out twice on separate occasions by the same technician for each participant. DXA abdominal fat measures showed excellent agreement with abdominal adipose tissue measured by MRI in a subset of the study population [48]. Scan artefacts were assessed by visual inspection. Scans with movement artefacts or with part of the body missing from the scan region were excluded from the analyses.

Weight was measured twice to the nearest 0.1 kg without shoes, using digital Seca scales (www.seca.com). Standing height was measured twice without shoes, using a portable stadiometer (Leicester height measure; Chasmors Ltd, Camden, London, UK). The average of the two values for each height measure was used in the analysis. BMI was calculated as weight(kg)/height(m)^2^.

### Statistical analysis

Participants were excluded from analyses if they did not have data on nutrient intake, physical activity, body fat, abdominal fat, height, or if there were major artefacts in the DXA measurements.

All analyses were conducted using STATA 11. An *a priori* decision was made to conduct analyses separately for the IMS and APCAPS populations because of their different age and anthropmetric profiles. Summary statistics were presented additionally stratified by sex. Variables were presented as means and standard deviations where normally distributed, and as geometric means (95% CI) or median (interquartile range) where skewed.

The outcome measures were total body fat (g), abdominal (L1 - L4) fat (g) and percentage abdominal fat (%)(calculated as L1 – L4 fat(g)/total body fat (g)). BMI (kg/m^2^) was also considered as an outcome variable, to see if there was any difference in associations when using BMI as a marker of body fat. The following dietary exposure variables were assessed: total energy intake (kcal/day), energy density (total energy intake/total weight of food consumed per day), and percent energy from fat, carbohydrate, and protein (using nutrient density method and adjusting for total energy intake). To ensure that the dietary results were robust to different methods of adjusting for energy intake, we also ran the analyses using the residuals method, whereby energy from each nutrient (protein/carbohydrate/fat) was regressed against energy intake from the two other nutrients [49]. The physical activity exposure variables were: total activity (METS-hrs/day), time spent in moderate/vigorous activity (mins/day) and time spent sedentary (mins/day). Linear regression models were used to examine associations between each of the outcome variables and diet/physicsal activity exposure variables. The first models adjusted for age and sex only, the final models adjusted for height, SLI and smoking (variables considered *a priori* to be potential confounders). Analyses of dietary exposures were additionally adjusted for total activity and analyses of physical activity measures were additionally adjusted for energy intake. Due to the sibling pair recruitment in the IMS study, robust standard errors were used to account for family clustering. Models were tested for interaction by sex using Likelihood Ratio Tests.

## Results

### Response

1962 participants from the IMS and 2601 participants from the APCAPS study were invited to join the HDS. Of the IMS participants invited, 918 (47%) were recruited. Of the APCAPS study, 1446 (56%) were recruited. This resulted in a total sample population of 2364. For these analyses, 156 participants were excluded because they did not have information on diet, body fat, abdominal fat, height, or there were major artefacts in the DXA measurements, leaving data on 2208 participants available for analysis (Figure 1).

### Study population characteristics

The two groups that formed the HDS population had quite different characteristics (Table 1). The IMS group were older (mean ages 51 and 47 years in men and women, range 20–79) than the APCAPS population (mean age of 21 in men and women, range 18–23). The APCAPS participants had low measures for all body fat markers: BMI, WHR, percent body fat, and percent abdominal body fat. While the APCAPS participants were at the lower end of normal with mean BMIs of around 19 kg/m^2^, the IMS men and women had mean BMIs of 24 and 26 kg/m^2^ respectively. Reported energy intake was higher in men than women, and within men it was higher in the APCAPS population than the IMS population. The APCAPS population had higher mean energy density, higher percent energy from carbohydrates, and lower percent energy from fat. The APCAPS participants also had higher reported levels of total and of moderate and vigorous physical activity than the IMS participants.

**Table 1:** **Study population characteristics by sex and original study recruited from, Hyderabad DXA study 2009-2010**

	**IMS**			**APCAPS**		
	**Males**	**Females**	**Total**	**Males**	**Females**	**Total**
**N**	447	382	829	964	415	1379
**Age**	50.5( 8.6)	47.2( 7.9)	49.0 (8.4)	20.8( 1.1)	21.0( 1.2)	20.8 (1.2)
**Weight (kg)**	67.3 (11.4)	62.4 (11.8)	65.1 (11.9)	54.8 (8.7)	44.4 (7.6)	51.6 (9.7)
**BMI†**	24.7( 3.7)	26.9( 4.7)	25.7 (4.3)	19.7( 2.8)	19.0( 2.9)	19.5 (2.8)
**WHR†**	0.94(0.06)	0.84(0.06)	0.89 (0.08)	0.82(0.04)	0.76(0.05)	0.80 (0.05)
**Total body fat (g)**	16724(5570)	23845(6881)	20006 (7149)	8025(7819,8237) ^†^	12141(11770,12523) ^†^	9090 (8885, 9299) ^†^
**Body fat (% of body weight)**	24.3 (4.9)	37.7 (5.0)	30.5 (8.3)	15.7 (5.0)	28.3 (5.3)	19.5 (7.7)
**Fat in L1L4 region (g)**	2480(1016)	2831(1098)	2642 (1069)	732( 708, 757) ^†^	895( 854, 937) ^†^	777 (756, 799) ^†^
**Percentage fat of L1L4 region (%)**	27.4( 6.7)	34.2( 6.8)	30.6 (7.6)	13.0(12.6,13.3) ^†^	19.8(19.1,20.4) ^†^	14.7 (14.4, 15.0) ^†^
**Percentage of total body fat in L1L4 region (%)**	14.5( 2.3)	11.6( 2.2)	13.1 (2.7)	9.3( 1.8)	7.5( 1.7)	8.8 (1.9)
**Height (cm)**	165.1( 6.2)	152.2( 5.7)	159.2 (8.8)	166.6( 6.3)	152.7( 5.2)	162.4 (8.8)
**Daily energy intake (kcal)**	2692( 824)	2023( 567)	2384 (791)	3288(1129)	2071( 632)	2922 (1150)
**Energy density (kcal/g)**	1.15(0.13)	1.10(0.14)	1.12 (0.14)	1.18(0.12)	1.19(0.18)	1.18 (0.14)
**Percentage of calories from fat (%)**	24.7( 5.5)	25.6( 5.5)	25.1 (5.5)	19.9( 5.6)	19.9( 6.1)	19.9 (5.8)
**Percentage of calories from protein (%)**	10.7( 1.1)	10.8( 1.1)	10.8 (1.1)	9.7( 0.9)	9.6( 0.9)	9.7 (0.9)
**Percentage of calories from carbohydrates (%)**	63.5( 6.5)	63.7( 6.1)	63.6 (6.3)	70.2( 6.1)	70.6( 6.5)	70.4 (6.2)
**Total METS (hrs/day)**	38.2( 5.7)	35.2( 5.2)	36.8 (5.6)	40.1( 6.5)	36.6( 5.3)	39.1 (6.4)
**Sedentary activity time (mins per day)**	380(169)	548(191)	457 (198)	367(203)	438 (188)	388 (201)
MVPA time (mins per day)*	160 (100, 254)	50 (26,93)	102 (47, 193)	196(116, 295)	70(35,135)	155 (77, 263)
**Standard of living index**	24.6 (6.1)	24.4 (6.7)	24.5 (6.4)	18.7(4.2)	17.7(4.5)	18.4 (4.3)

METS = metabolic equivalent of a task, representing energy expenditure per day; MVPA = moderate/vigorous physical activity.

Values represent means (SD) unless otherwise stated.

†Geometric means ( 95% CI).

*Median (IQR).

p < 0.001 for difference between IMS and APCAPS total populations, for all variables.

There was weak evidence for an interaction (p = 0.03) between sex and only one of the exposure variables (total physical activity) so data from the two sexes were combined for the main analyses. For results stratified by sex, see the Additional file 1.

### Body fat and diet associations

In the APCAPS population, energy intake was positively associated with body fat. A 100 kcal higher energy intake was associated with 45 g higher body fat (95% CI 22, 68). There was no strong evidence of association with any of the dietary components investigated following adjustments for possible confounders (Table 2). In contrast, in the IMS population, there was no evidence for an association between energy intake and body fat (17 g, 95% CI −40,73), but associations with dietary components were identified. Protein intake was positively associated with body fat: after adjustment for total energy a 1% higher proportion of energy from protein was associated with 509 g (137, 881) higher total body fat. In the age and sex adjusted models there was evidence of positive association between fat intake and body fat and inverse association between carbohydrate intake and body fat, but these associations were attenuated in the final model.

**Table 2:** **Associations between total body fat (grams), and diet and physical activity variables [β coefficients (95% CI)], by original study recruited from, Hyderabad DXA Study 2009-2010**

	**IMS (N = 829)**				**APCAPS (N = 1379)**			
**Exposure variable**	**Age & sex adjusted**	**p-value**	**Final model**	**p-value**	**Age & sex adjusted**	**p-value**	**Final model**	**p-value**
**Daily energy intake (per 100 kcal)**	49.8 (−12.4,112.0)	0.12	16.8 (−39.5,73.1)	0.56	54.3 (31.8,76.7)	<0.001	44.9 (21.5,68.3)	<0.001
**Energy density (kcal/g)**	2219.7 (−1397.4,5836.7)	0.23	1281.2 (−1978.4,4540.7)	0.44	810.3 (−569.8,2190.4)	0.25	614.9 (−761.7,1991.4)	0.38
**Percentage of calories from protein (%)**	966.7 (605.4,1328.0)	<0.001	508.8 (136.6,880.9)	0.01	303.9 (56.6,551.3)	0.02	227.4 (−11.4,466.3)	0.06
**Percentage of calories from fat (%)**	168.3 (88.0,248.5)	<0.001	59.6 (−16.1,135.4)	0.12	27.8 (−9.9,65.5)	0.15	15.6 (−20.4,51.5)	0.40
**Percentage of calories from carbohydrates (%)**	−150.9 (−221.1,-80.7)	<0.001	−62.4 (−127.1,2.3)	0.06	−32.2 (−67.1,2.7)	0.07	−21.0 (−54.3,12.3)	0.22
**Total METS (hrs/day)**	−236.9 (−314.0,-159.9.1)	<0.001	−145.3 (−217.8,-72.8)	<0.001	−46.9 (−80.5,-13.2)	0.01	−46.3 (−80.8,-11.8)	0.01
**Time spent sedentary (per 10 mins/day)**	29.3(2.3,56.3)	0.03	25.5 (0.1,51.0)	0.05	3.0 (−7.5,13.5)	0.57	4.3 (−6.4,15.0)	0.43
**MVPA time (per 10 mins/day)**	−114.2 (−145.8,-82.5)	<0.001	−63.2 (−95.1,-31.3)	<0.001	−29.3 (−43.7,-14.9)	<0.001	−28.2 (−43.2,-13.3)	<0.001

METS = metabolic equivalent of a task, representing energy expenditure per day; MVPA = moderate/vigorous physical activity.

Final model adjusted for age, sex, smoking, SLI, height.

Diet variables adjusted for total METS, percentage diet variables additionally adjusted for total energy intake.

PA variables adjusted for energy intake.

Robust standard errors used to account for family clustering.

The associations found between diet and abdominal body fat (g) were broadly the same as found for total body fat (Table 3). When considering the *proportion* of fat located in the abdominal region, the same dietary variables were found to be important but the effect sizes were smaller (Table 4). In the APCAPS population, each 100 kcal higher daily energy intake was associated with 0.02% (0.01, 0.03) higher proportion of fat distributed abdominally. In the IMS population there was no association with total energy intake. However, percent protein and fat were positively associated with proportion of fat distributed in the abdominal region (0.18% (0.05, 0.32) and 0.03% (0.01, 0.06) higher proportion of fat in the abdominal region per 1% higher proportion of energy from protein and fat respectively). Percent carbohydrate was inversely associated with abdominal fat (0.04% (0.06, 0.02) decrease in proportion of fat distributed in the abdominal region per 1% higher proportion of energy from carbohydrate).

**Table 3:** **Associations between abdominal body fat (L1 to L4, grams), and diet and physical activity variables [β coefficients (95% CI)], by original study recruited from, Hyderabad DXA Study 2009-2010**

	**IMS (N = 829)**				**APCAPS (N = 1379)**			
**Exposure variable**	**Age, sex adjusted**	**p-value**	**Final model**	**p-value**	**Age, sex adjusted**	**p-value**	**Final model**	**p-value**
**Daily energy intake (per 100 kcal)**	4.7 (−5.6,14.9)	0.37	1.4 (−8.1,10.8)	0.78	7.1 (4.1,10.0)	<0.001	6.2 (3.1,9.3)	<0.001
**Energy density (kcal/g)**	297.5 (−255.7,850.6)	0.29	170.4 (−333.1,673.8)	0.51	105.5 (−65.2,276.2)	0.23	73.9 (−97.0,244.7)	0.40
**Percentage of calories from protein (%)**	169.7 (108.9,230.5)	<0.001	99.1 (35.9,162.3)	<0.001	38.3 (6.3,70.4)	0.02	30.1 (−1.5,61.7)	0.06
**Percentage of calories from fat (%)**	30.7 (17.3,44.2)	<0.001	13.8 (0.6,27.0)	0.04	2.6 (−2.2,7.5)	0.29	1.1 (−3.6,5.8)	0.65
**Percentage of calories from carbohydrates (%)**	−28.3 (−39.8,-16.8)	<0.001	−14.8 (−26.0,-3.7)	0.01	−3.2 (−7.7,1.3)	0.16	−1.8 (−6.2,2.6)	0.42
**Total METS (hrs/day)**	−40.7 (−53.4,-27.9)	<0.001	−26.9 (−39.6,-14.1)	<0.001	−3.5 (−8.0,1.0)	0.12	−3.6 (−8.2,1.0)	0.13
**Time spent sedentary (per 10 mins)**	4.8 (0.3,9.4)	0.04	4.2 (−0.2,8.6)	0.06	−0.3 (−1.6,1.1)	0.68	0.0 (−1.4,1.4)	0.96
**MVPA time (per 10 mins)**	−18.6 (−24.0,-13.2)	<0.001	−10.9 (−16.7,-5.2)	<0.001	−2.8 (−4.7,-0.9)	0.004	−2.8 (−4.8,-0.8)	0.01

METS = metabolic equivalent of a task, representing energy expenditure per day; MVPA = moderate/vigorous physical activity.

Final models adjusted for age, sex, smoking, SLI.

Diet variables adjusted for total METS, percentage diet variables additionally adjusted for total energy intake.

PA variables adjusted for energy intake.

Robust standard errors used to account for family clustering.

**Table 4:** **Associations between% fat in the abdominal region (L1 to L4), and diet and physical activity variables [β coefficients (95% CI)], by original study recruited from, Hyderabad DXA Study 2009-2010**

	**IMS (N = 829)**				**APCAPS (N = 1379)**			
**Exposure variable**	**Age & sex adjusted**	**p-value**	**Final model**	**p-value**	**Age & sex adjusted**	**p-value**	**Final model**	**p-value**
**Daily energy intake (per 100 kcal)**	−0.007 (−0.028,0.014)	0.51	−0.003 (−0.023,0.018)	0.81	0.018 (0.009,0.028)	<0.001	0.019 (0.010,0.028)	<0.001
**Energy density (kcal/g)**	−0.082 (−1.123,0.959)	0.88	−0.120 (−1.107,0.867)	0.81	0.441 (−0.091,0.972)	0.10	0.350 (−0.188,0.888)	0.20
**Percentage of calories from protein (%)**	0.292 (0.162,0.421)	<0.001	0.180 (0.045,0.315)	0.01	0.047 (−0.053,0.148)	0.36	0.035 (−0.067,0.137)	0.50
**Percentage of calories from fat (%)**	0.061 (0.034,0.089)	<0.001	0.034 (0.006,0.062)	0.02	0.003 (−0.013,0.019)	0.69	−0.0004 (−0.017,0.016)	0.96
**Percentage of calories from carbohydrates (%)**	−0.059 (−0.082,-0.035)	<0.001	−0.039 (−0.063,-0.015)	0.002	−0.004 (−0.019,0.011)	0.61	−0.0004 (−0.016,0.015)	0.96
**Total METS (hrs/day)**	−0.067 (−0.097,-0.038)	<0.001	−0.048 (−0.078,-0.019)	0.001	0.001 (−0.014,0.016)	0.94	−0.001 (−0.016,0.014)	0.94
**Time spent sedentary (per 10 mins)**	0.008 (−0.001,0.017)	0.08	0.007 (−0.002,0.016)	0.12	−0.003 (−0.008,0.001)	0.15	−0.002 (−0.007,0.003)	0.37
**MVPA time (per 10 mins)**	−0.030 (−0.043,-0.017)	<0.001	−0.019 (−0.033,-0.006)	0.01	−0.004 (−0.010,0.003)	0.29	−0.004 (−0.011,0.002)	0.22

METS = metabolic equivalent of a task, representing energy expenditure per day; MVPA = moderate/vigorous physical activity.

Final model model adjusted for age, sex, smoking, SLI, height.

Diet variables adjusted for total METS, percentage diet variables additionally adjusted for total energy intake.

PA variables adjusted for energy intake.

Robust standard errors used to account for family clustering.

### Body fat and physical activity associations

Total activity (MET-hrs/day) and time (min/day) spent in moderate/vigorous physical activity were inversely proportional to total body fat in both studies. Associations remained significant following adjustment for confounders (Table 2). In the APCAPS cohort, one MET-hour higher activity was associated with 46 g (95% CI 12, 81) less body fat; in the IMS with 145 g less body fat (95% CI 73, 218). Ten minutes more MVPA time per day was associated with 62 g (95% CI 31, 95) less total fat in the IMS and 28 g (95% CI 14, 43) less fat in the APCAPS samples. There was some evidence for a small positive association between sedentary time and total fat in the IMS (an extra 10 minutes of time spent sedentary per day was associated with 26 g higher body fat (95% CI 0.1, 51)), but no association was observed in APCAPS. When using the residuals method as an alternative way to adjust for energy intake, results were materially unchanged.

The associations found between physical activity and abdominal body fat (g) were broadly the same as found for total body fat (Tables 3 and 4). There was no evidence for associations between physical activity and distribution of body fat in the APCAPS population. In the IMS population one more MET-hour per day was associated with a 0.05% (95% CI 0.02, 0.08) lower proportion of body fat in the abdominal region, and 10 minutes more moderate/vigorous activity with a 0.02% (95% CI 0.01, 0.03) lower proportion of body fat in the abdominal region.

### BMI and diet/physical activity associations

When BMI was used as the outcome instead of total body fat, the same associations were found with most exposures (Table 5). The exception was physical activity in the APCAPS population. Total physical activity and moderate/vigorous physical activity were not associated with BMI in the APCAPS population, although an association had been identified with total body fat.

**Table 5:** **Associations between BMI(kg/m** ^**2**^ **), and diet and physical activity variables [β coefficients (95% CI)], by original study recruited from, Hyderabad DXA Study 2009-2010**

	**IMS (N = 829)**				**APCAPS (N = 1379)**			
	**Age, sex adjusted**	**p-value**	**Final model**	**p-value**	**Age, sex adjusted**	**p-value**	**Final model**	**p-value**
**Daily energy intake (per 100 kcal)**	0.033 (−0.008,0.075)	0.11	0.025 (−0.013,0.063)	0.20	0.050 (0.034,0.065)	<0.001	0.044 (0.028,0.060)	<0.001
**Energy density (kcal/g)**	1.756 (−0.802,4.313)	0.18	1.45 (−0.89,3.79)	0.23	0.561 (−0.456,1.578)	0.28	0.269 (−0.783,1.321)	0.62
**Percentage of calories from protein (%)**	0.712 (0.464,0.959)	<0.001	0.388 (0.128,0.648)	0.004	0.151 (−0.014,0.316)	0.07	0.115 (−0.048,0.278)	0.17
**Percentage of calories from fat (%)**	0.130 (0.078,0.183)	<0.001	0.054 (0.002,0.106)	0.04	0.005 (−0.021,0.031)	0.72	−0.003 (−0.029,0.022)	0.80
**Percentage of calories from carbohydrates (%)**	−0.119 (−0.165,-0.074)	<0.001	−0.056 (−0.101,-0.012)	0.01	−0.008 (−0.032,0.016)	0.50	−0.001 (−0.025,0.023)	0.94
**Total METS (hrs/day)**	−0.135 (−0.186,-0.083)	<0.001	−0.081 (−0.131,-0.032)	0.001	0.010 (−0.013,0.033)	0.39	0.007 (−0.016,0.030)	0.55
**Time spent sedentary (per 10 mins)**	0.020 (0.002,0.038)	0.03	0.019 (0.001,0.036)	0.03	−0.003 (−0.010,0.004)	0.44	−0.000 (−0.007,0.007)	0.97
**MVPA time (per 10 mins)**	−0.066 (−0.088,-0.045)	<0.001	−0.036 (−0.057,-0.014)	0.001	−0.004 (−0.015,0.006)	0.42	−0.005 (−0.016,0.005)	0.33

METS = metabolic equivalent of a task, representing energy expenditure per day; MVPA = moderate/vigorous physical activity.

Final models were adjusted for age,sex, smoking, SLI.

Diet variables adjusted for total METS, percentage diet variables additionally adjusted for total energy intake.

PA variables adjusted for energy intake.

Robust standard errors used to account for family clustering.

### Sensitivity analyses

The inclusion of spouse pairs in the IMS population could have introduced some statistical dependency and influenced results. A sensitivity analysis was done, removing a random member of each spouse pair from the analysis, and the results were unchanged. In addition, in the APCAPS sample the residuals from the regression were slightly skewed. A further sensitivity analysis using the transformed variables showed that the results were materially unchanged. In addition, to ensure that the results were robust to different indices of body fat we re-ran the analyses using two alternative indices: percent body fat and fat mass index (fat/height-squared), and found that the results were materially unchanged.

## Discussion

Higher levels of physical activity were related to lower levels of body fat in both cohorts. The associations between dietary variables and total body fat varied between the cohorts: in the APCAPS population higher total energy intake was related to higher total body fat, whereas in the IMS population associations were found with the composition of the diet. The patterns of relationships with abdominal adiposity closely matched those of total body fat.

Overall, the APCAPS population (in contrast to the IMS population) showed no association between physical activity and proportion of fat in the abdominal region and no evidence of any associations between body fat and dietary constituents. This could be because the effects of diet and activity on body fat distribution are the result of cumulative exposure to established patterns over time. In particular, fat becomes more centrally distributed as people get older [50-52]. The APCAPS population may therefore be too young (mean age 21) to detect the associations that are seen in the older IMS population. While such associations have been seen in children in western populations [53,54], India is in an earlier stage of the nutrition transition. This may be something that develops later on in India as diets become even more ‘obesogenic’. If this is the case it highlights the importance of studying the effects of diet and activity across different ages and looking at how long term patterns are established across the life course.

### Comparison with previous research

The body of research on associations between dietary factors and body fat is mixed. Both positive and inverse associations have been found between energy intake and body fat; this analysis was consistent with studies showing positive associations [18,55,56], while an inverse association is also often found and attributed to more active participants having higher energy intakes [49]. The same mixed picture is seen for protein [19-21], with only some of these studies finding the positive association as seen here (in the IMS sample) [19,21]. The associations of higher body fat with higher dietary fat and lower dietary carbohydrate are consistent with some other studies [15,17,18,21], although there are again mixed findings in this area, with some studies finding no effect [16,19].

The associations found between physical activity and body fat are consistent with other research, and biologically plausible [57-59]. The impact on obesity was most apparent for total physical activity (MET-hrs/day) and for time spent in moderate/vigorous activity. Some evidence points to moderate and vigorous physical activity being the most beneficial intensity of activity, although mainly with respect to chronic disease outcomes rather than body fat itself [60]. Some studies have shown associations between body fat and sedentary time [33-35,61], but only a weak association among the older participants was seen here. With all measures of physical activity (and diet) considered, there is a problem with inferring causality when many of the studies (including this one) are cross-sectional. While it is often assumed that lower levels of physical activity lead to obesity, there is also evidence for a reverse effect – that obesity leads to reduced levels of activity, and the association may be bidirectional, potentially to different degrees in different populations [61].

In the APCAPS population there was no evidence of association between physical activity and BMI or abdominal adiposity (in contrast to physical activity and body fat). There are other studies which have similarly found no association between physical activity and abdominal obesity in young people [62]. Studies have also found less evidence of association between physical activity and obesity when using BMI as an outcome compared with body fat [53,63]. One possible explanation for this is that physical activity promotes fat loss while maintaining or increasing lean tissue mass. This would mean that the benefits of physical activity are apparent when measuring body fat directly, but not when using BMI as a surrogate marker because the lean mass contributes to weight.

### Strengths and limitations

Strengths of the study include the incorporation of both men and women from rural and urban areas, and the wide age range of participants. The study sample was large, and was on an Indian population that has relatively few prior studies on this subject. The DXA measurements provided an accurate measure of adiposity, and there were detailed measurements of both diet and physical activity.

Due to the cross-sectional design of the study we cannot infer causality. FFQs tend to over-estimate consumption, so the absolute effect sizes found for beta coefficients should be interpreted with caution [49]. The physical activity measures may also have overestimated activity, particularly moderate and vigorous activities [43]. This bias would be most problematic if the overestimations were associated with the outcome of body fat (i.e. systematic), however, in this study body fat was measured objectively through DXA scanning.

## Conclusions

In this Indian population, physical activity variables were the exposures most consistently associated with body fat. Dietary variables are also important, with a higher fat and protein and lower carbohydrate diet associated with higher body fat. However, this association was not found consistently across populations. The same diet and activity variables may be important in determining distribution of body fat as well as total overall amount of body fat.

The different associations seen in the two Indian populations suggest that it is important to take age into account and to study the effects of diet and activity on body fat across the life course. Further longitudinal research is needed to indicate causality and directions of associations.

## Additional file

Additional file 1:
**Although there was no strong evidence found for any interaction between sex and the exposure variables, as there are important differences in body fat by sex we have included results stratified by sex for reference below.** The same pattern of results was seen when data are separated by sex, although there were smaller numbers of females and therefore reduced statistical power in these groups.

## Abbreviations

IMS: Indian Migration Study
APCAPS: Andhra Pradesh Children and Parents’ Study
HDS: Hyderabad DXA Study
PA: Physical activity
SLI: Standard of Living Index
FFQ: Food frequency questionnaire
USDA: United States Department of Agriculture
MET: Metabolic equivalent of a task
IEI: Integrated energy index
