# Supplementary material for: Associations between diet, physical activity and body fat distribution: a cross sectional study in an Indian population
Source: BMC Public Health. 2015 Mar 24;15:281. doi: 10.1186/s12889-015-1550-7 (PMC4381479; doi:10.1186/s12889-015-1550-7)
Supplement: Additional file 1: — Although there was no strong evidence found for any interaction between sex and the exposure variables, as there are important differences in body fat by sex we have included results stratified by sex for reference below. The same pattern of results was seen when data are separated by sex, although there were smaller numbers of females and therefore reduced statistical power in these groups. [file 12889_2015_1550_MOESM1_ESM.doc]

**Table 1** Associations between total body fat (grams), and diet and physical activity variables [β coefficients (95% CI)] in IMS population, by sex, Hyderabad DXA Study 2009-2010

|  | IMS males (N = 447) | | | | IMS females (N = 382) | | | |
| --- | --- | --- | --- | --- | --- | --- | --- | --- |
|  | Age adjusted | p-value | Final model | p-value | Age adjusted | p-value | Final model | p-value |
| Daily energy intake (per 100 kcal) | 60.8 (−8.7, 130,2) | 0.09 | 55.7 (−5.5, 116.8) | 0.07 | 28.3 (−102.1, 158.8) | 0.67 | −72.4 (−188.2, 43.4) | 0.2 |
| Energy density (kcal/g) | 4056.8 (−333.9, 8447.5) | 0.07 | 3194.5 (−498.4, 6887.4) | 0.09 | 404.1 (−5311.1, 6119.3) | 0.89 | −576.4 (−5883.7, 4730.9) | 0.83 |
| Percentage of calories from protein (%)† | 1132.1 (693.5, 1570.7) | <0.001 | 669.9 (253.2, 1086.7) | 0.002 | 779.4 (184.2, 1374.6) | 0.01 | 336.2 (−302.3, 974.8) | 0.3 |
| Percentage of calories from fat (%)† | 191.2 (93.3, 289.1) | <0.001 | 85.3 (−7.6, 178.2) | 0.07 | 147.7 (15.9, 279.5) | 0.03 | 40.8 (−81.5, 163.1) | 0.5 |
| Percentage of calories from carbohydrates (%)† | −157.5 (−241.9, −73.2) | <0.001 | −80.7 (−156.1, −5.4) | 0.03 | −146.0 (−262.5, −29.5) | 0.01 | −44.4 (−156.1, 67.3) | 0.4 |
| Total METS (hrs/day) | −248.8 (−336.1, −161.5) | <0.001 | −164.7 (−251.4, −78.1) | <0.001 | −221.2 (−363.1, −79.3) | 0.002 | −132.3 (−256.0, −8.5) | 0.04 |
| Sedentary activity time (per 10 mins) | 15.5 (−17.9, 49.0) | 0.36 | 28.6 (−2.7, 59.8) | 0.07 | 41.2 (0.3, 82.0) | 0.048 | 22.2 (−17.6, 62.0) | 0.3 |
| MVPA time (per 10 mins) | −110.4 (−143.2, −77.5) | <0.001 | −67.4 (−102.3, −32.5) | <0.001 | −121.7 (−191.5, −52.0) | 0.001 | −65.7 (−129.1, −2.2) | 0.04 |

**Table 2** Associations between total body fat (grams), and diet and physical activity variables [β coefficients (95% CI)] in APCAPS population, by sex, Hyderabad DXA Study 2009-2010

|  | APCAPS males (N = 964) | | | | APCAPS females (N = 415) | | | |
| --- | --- | --- | --- | --- | --- | --- | --- | --- |
|  | Age adjusted | p-value | Final model | p-value | Age adjusted | p-value | Final model | p-value |
| Daily energy intake (per 100 kcal) | 50.7 (26.5, 74.9) | <0.001 | 44.8 (19.5, 70.1) | 0.001 | 80.9 (16.5, 145.3) | 0.01 | 51.8 (−16.4, 120.0) | 0.1 |
| Energy density (kcal/g) | 737.1 (−1263.4, 2737.7) | 0.50 | 361.3 (−1624.3, 2347.0) | 0.72 | 890.6 (−881.9, 2663.2) | 0.3 | 823.0 (−1147.7, 2793.7) | 0.4 |
| Percentage of calories from protein (%)† | 278.2 (−9.8, 566.2) | 0.06 | 242.4 (−44.0, 528.8) | 0.10 | 375.4 (−98.5, 849.4) | 0.1 | 145.4 (−281.1, 571.9) | 0.5 |
| Percentage of calories from fat (%)† | 7.91 (−35.6, 51.5) | 0.70 | 3.0 (−40.0, 46.1) | 0.90 | 65.6 (−0.0, 131.3) | 0.05 | 24.3 (−37.6, 86.2) | 0.4 |
| Percentage of calories from carbohydrates (%)† | −16.1 (−56.1, 23.7) | 0.40 | −12.5 (−52.1, 27.2) | 0.50 | −64.2 (−126.8, −1.6) | 0.05 | −24.1 (−82.3, 34.1) | 0.4 |
| Total METS (hrs/day) | 19.4 (−59.0, 17.1) | 0.30 | −28.6 (−67.7, 10.4) | 0.15 | −139.2 (−206.4, −72.0) | <0.001 | −102.5 (−172.7, −32.3) | 0.004 |
| Sedentary activity time (per 10 mins) | −5.4 (−17.4, 6.7) | 0.4 | −1.3 (−13.9, 11.3) | 0.83 | 25.9 (6.0, 45.7) | 0.01 | 17.7 (−2.6, 38.0) | 0.09 |
| MVPA time (per 10 mins) | −20.9 (−37.6, −4.2) | 0.01 | −23.3 (−40.4, −6.1) | 0.01 | −55.5 (−81.7, −29.3) | <0.001 | −42.4 (−73.0, −11.9) | 0.007 |

METS = metabolic equivalent of a task, representing energy expenditure per day; MVPA = moderate/vigorous physical activity.

Final model adjusted for age, smoking, SLI, height.

Diet variables adjusted for total METS, percentage diet variables additionally adjusted for total energy intake.

PA variables adjusted for energy intake.

Robust standard errors used to account for family clustering.

**Table 3** Associations between% fat in the abdominal region (L1 to L4), and diet and physical activity variables [β coefficients (95% CI)] in the IMS population, by sex, Hyderabad DXA Study 2009-2010

|  | IMS males | | | | IMS FEMALES | | | |
| --- | --- | --- | --- | --- | --- | --- | --- | --- |
|  | Age adjusted | p-value | Final model | p-value | Age adjusted | p-value | Final model | p-value |
| Daily energy intake (per 100 kcal) | 0.0002 (−0.025, 0.025) | 0.90 | 0.004 (−0.021, 0.029) | 0.74 | −0.027 (−0.063, 0.008) | 0.13 | −0.024 (−0.060, 0.013) | 0.2 |
| Energy density (kcal/g) | 0.654 (−0.886, 2.192) | 0.40 | 0.621 (−0.756, 1.999) | 0.38 | −0.801 (−2.297, 0.695) | 0.29 | −0.853 (−2.303, 0.598) | 0.25 |
| Percentage of calories from protein (%)† | 0.425 (0.243, 0.607) | <0.001 | 0.259 (0.074, 0.444) | 0.006 | 0.148 (−0.032, 0.328) | 0.11 | 0.087 (−0.107, 0.282) | 0.38 |
| Percentage of calories from fat (%)† | 0.089 (0.049, 0.128) | <0.001 | 0.050 (0.010, 0.089) | 0.01 | 0.034 (−0.006, 0.075) | 0.09 | 0.022 (−0.020, 0.063) | 0.31 |
| Percentage of calories from carbohydrates (%)† | −0.081 (−0.114, −0.048) | <0.001 | −0.054 (−0.087, −0.021) | 0.001 | −0.033 (−0.067, 0.002) | 0.06 | −0.021 (−0.057, 0.015) | 0.26 |
| Total METS (hrs/day) | −0.076 (−0.114, −0.038) | <0.001 | −0.044 (−0.082, −0.007) | 0.02 | −0.055 (−0.100, −0.010) | 0.02 | −0.048 (−0.094, −0.003) | 0.04 |
| Sedentary activity time (per 10 mins) | 0.006 (−0.007, 0.019) | 0.34 | 0.010 (−0.001, 0.023) | 0.09 | 0.009 (−0.003, 0.022) | 0.13 | 0.007 (−0.006, 0.020) | 0.27 |
| MVPA time (per 10 mins) | −0.034 (−0.050, −0.018) | <0.001 | −0.018 (−0.034, −0.001) | 0.03 | −0.022 (−0.045, 0.001) | 0.06 | −0.018 (−0.042, 0.005) | 0.12 |

**Table 4** Associations between% fat in the abdominal region (L1 to L4), and diet and physical activity variables [β coefficients (95% CI)] in the APCAPS population, by sex, Hyderabad DXA Study 2009-2010

|  | APCAPS males | | | | APCAPS FEMALES | | | |
| --- | --- | --- | --- | --- | --- | --- | --- | --- |
|  | Age adjusted | p-value | Final model | p-value | Age adjusted | p-value | Final model | p-value |
| Daily energy intake (per 100 kcal) | 0.017 (0.007, 0.028) | 0.001 | 0.018 (0.008, 0.028) | 0.001 | 0.024 (−0.001, 0.048) | 0.06 | 0.025 (0.001, 0.050) | 0.05 |
| Energy density (kcal/g) | 0.581 (−0.238, 1.399) | 0.16 | 0.331 (−0.503, 1.165) | 0.43 | 0.278 (−0.408, 0.965) | 0.42 | 0.339 (−0.354, 1.031) | 0.34 |
| Percentage of calories from protein (%)† | 0.063 (−0.057, 0.184) | 0.3 | 0.047 (−0.074, 0.169) | 0.45 | 0.013 (−0.164, 0.190) | 0.88 | 0.013 (−0.167, 0.194) | 0.89 |
| Percentage of calories from fat (%)† | 0.001 (−0.025, 0.027) | 0.95 | −0.001 (−0.021, 0.020) | 0.95 | 0.001 (−0.025, 0.027) | 0.95 | 0.001 (−0.025, 0.028) | 0.92 |
| Percentage of calories from carbohydrates (%)† | −0.006 (−0.025, 0.013) | 0.53 | −0.000 (−0.019, 0.018) | 0.92 | −0.001 (−0.026, 0.024) | 0.93 | −0.002 (−0.027, 0.024) | 0.9 |
| Total METS (hrs/day) | 0.004 (−0.013, 0.021) | 0.64 | 0.002 (−0.015, 0.019) | 0.78 | −0.007 (−0.039, 0.025) | 0.68 | −0.007 (−0.040, 0.026) | 0.67 |
| Sedentary activity time (per 10 mins) | −0.004 (−0.010, 0.001) | 0.13 | −0.002 (−0.008, 0.003) | 0.38 | −0.001 (−0.010, 0.008) | 0.83 | −0.001 (−0.010, 0.009) | 0.89 |
| MVPA time (per 10 mins) | −0.002 (−0.010, 0.006) | 0.61 | −0.003 (−0.011, 0.005) | 0.46 | −0.009 (−0.021, 0.004) | 0.18 | −0.009 (−0.021, 0.004) | 0.19 |

METS = metabolic equivalent of a task, representing energy expenditure per day; MVPA = moderate/vigorous physical activity.

Final model model adjusted for age, smoking, SLI, height.

Diet variables adjusted for total METS, percentage diet variables additionally adjusted for total energy intake.

PA variables adjusted for energy intake.

Robust standard errors used to account for family clustering.
